# Optical Coherence Tomography-Guided Early Versus Late Switching to Dexamethasone Implants in Macular Edema Related to Central Retinal Vein Occlusion: Real-World Evidence

**DOI:** 10.3390/diagnostics15040439

**Published:** 2025-02-11

**Authors:** Zübeyir Yozgat

**Affiliations:** Department of Ophthalmology, Kastamonu University, Kastamonu 37150, Turkey; zyozgat@kastamonu.edu.tr

**Keywords:** central retinal vein occlusion, macular edema, optical coherence tomography, anti-VEGF therapy, dexamethasone implant, real-world study

## Abstract

**Background/Objectives:** This study evaluated the outcomes of early versus late switching from intravitreal anti-vascular endothelial growth factor (anti-VEGF) therapy to dexamethasone (DEX) implants in patients with macular edema secondary to central retinal vein occlusion (CRVO). The critical role of optical coherence tomography (OCT) in guiding therapeutic decisions and monitoring treatment responses is emphasized. **Methods:** In this real-world, retrospective study, 61 treatment-naïve CRVO patients were divided into two groups: Group 1 (early switch after three anti-VEGF injections) and Group 2 (late switch after six months of anti-VEGF therapy). High-resolution OCT was employed at all follow-ups to evaluate anatomical outcomes, specifically changes in central macular subfield thickness (CMST), while best-corrected visual acuity (BCVA) was assessed using standardized ETDRS charts. **Results:** Both groups demonstrated significant improvements in BCVA and reductions in CMST over 52 weeks. Group 1 exhibited slightly greater gains in BCVA (+20.3 ETDRS letters) and a greater CMST reduction (−201.5 µm) compared to Group 2 (+18.5 ETDRS letters, −184.4 µm), although the differences were not statistically significant. The OCT findings enabled precise monitoring and individualized treatment adjustments, reducing the treatment burden in the early-switch group with fewer anti-VEGF injections. **Conclusions:** Early switching to DEX implants, guided by OCT findings, may optimize therapeutic outcomes and reduce the treatment burden, particularly in real-world settings with limited resources or adherence challenges. These findings highlight the importance of incorporating advanced imaging techniques into routine practice, and underscore the need for further research on OCT-guided therapeutic transitions in macular edema management.

## 1. Introduction

Retinal vein occlusion (RVO) is the second most common retinal vascular disorder after diabetic retinopathy, encompassing two main types: branch retinal vein occlusion (BRVO) and central retinal vein occlusion (CRVO) [1]. BRVO occurs when a branch of the central retinal vein becomes obstructed, typically affecting a localized segment of the retina. In contrast, CRVO arises from an obstruction of the central retinal vein at or near the lamina cribrosa, leading to widespread retinal ischemia and edema.

CRVO is further classified into ischemic and non-ischemic forms, with the ischemic subtype carrying a higher risk of complications, such as neovascular glaucoma and severe visual loss [2]. Macular edema, the most frequent cause of vision impairment in CRVO, results from increased vascular permeability and leakage [3]. The effective management of macular edema is crucial for preserving vision and reducing the burden of retinal complications. Macular edema is the principal cause of visual impairment in CRVO, and remains a challenging condition to treat effectively [4].

Recent advances in retinal imaging and intravitreal therapeutics have significantly improved management outcomes, with anti-vascular endothelial growth factor (anti-VEGF) agents and intravitreal corticosteroids representing the primary treatment modalities. Ischemia plays a pivotal role in the pathophysiology of CRVO, particularly in its ischemic subtype, by leading to retinal hypoxia and the upregulation of VEGF. This hypoxic environment triggers pathological angiogenesis as a compensatory response to restore oxygen supply, which in turn contributes to increased vascular permeability and macular edema [3,5]. Anti-VEGF agents target this pathological angiogenesis and vascular leakage associated with CRVO [6]. These treatments, such as ranibizumab and aflibercept, have demonstrated excellent efficacy in reducing macular thickness and improving visual acuity [5,7]. However, their short duration of action often necessitates frequent injections that are typically administered every four to six weeks, imposing a considerable treatment burden on patients [5,7]. Studies have indicated that patients receiving anti-VEGF therapy for CRVO-related macular edema require an average of seven to nine injections per year to maintain visual and anatomical benefits [5,7,8].

Intravitreal dexamethasone (DEX) implants provide an alternative, offering sustained-release corticosteroid therapy that effectively reduces inflammation and vascular permeability [9,10,11]. The DEX implant has shown particular promise in patients with suboptimal responses to anti-VEGF or those with contraindications to frequent injections [12,13,14]. Studies have highlighted its ability to reduce the central macular thickness and maintain visual gains with fewer injections compared to anti-VEGF therapies [4,10,15,16]. Intravitreal DEX implants (Ozurdex) offer a longer duration of action compared to anti-VEGF agents, typically requiring reinjection every 4 to 6 months, depending on the individual patient’s response and recurrence of macular edema [10,15]. Clinical studies indicate that most patients require an average of two to three injections per year to maintain therapeutic effects [16,17].

Optical coherence tomography (OCT) has revolutionized the diagnosis and management of macular diseases, including macular edema secondary to retinal vein occlusion. Its high-resolution imaging capabilities provide precise assessments of the retinal layers, enabling the early detection of structural changes and guiding therapeutic decisions. OCT’s role extends to monitoring the treatment response, as it can quantitatively evaluate the central macular thickness and detect subtle anatomical variations that might influence clinical outcomes. Studies have shown that multimodal imaging, including OCT and OCT angiography, improves diagnostic accuracy and supports personalized treatment approaches, particularly for complex conditions such as retinal vein occlusion [18,19]. Furthermore, emerging applications, such as artificial intelligence, in OCT imaging are set to enhance the precision of diagnoses and prognoses for rare macular edema subtypes [20].

Despite these advancements, the optimal timing for transitioning between anti-VEGF and DEX implant therapies remains under debate. Early switching may reduce the treatment burden and prevent chronic edema, while a delayed transition could preserve the anti-VEGF benefits before introducing corticosteroids. Understanding the balance among efficacy, safety, and patient-specific factors is crucial for optimizing outcomes in CRVO management.

To address these gaps, this study aimed to compare the outcomes of early versus late switching from anti-VEGF therapy to DEX implants in patients with macular edema secondary to CRVO, with a particular focus on the role of OCT in guiding these therapeutic decisions. By leveraging OCT’s ability to provide high-resolution imaging and a quantitative analysis of the central macular thickness, this study sought to enhance the understanding of the timing and effectiveness of these therapeutic approaches. The findings highlight OCT’s potential as a critical tool for optimizing personalized treatment strategies and improving patient outcomes in real-world clinical settings.

## 2. Materials and Methods

### 2.1. Study Design and Patients

This comparative, observational, real-world study included 61 eyes of 61 treatment-naïve patients with macular edema secondary to CRVO. The data, which included high-resolution OCT scans, were extracted from the hospital’s patient archive records, and this study was conducted between January 2021 and December 2023.

The patients were divided into two groups, based on the timing of switching from intravitreal anti-VEGF therapy to DEX implants:

- Group 1 (early switch, *n* = 31);
- Group 2 (late switch, *n* = 30).

This study adhered to the tenets of the Declaration of Helsinki and was approved by the local ethics committee (protocol code: 2024-KAEK-124; date of approval: 10 December 2024).

Informed consent was obtained from all participants.

### 2.2. Inclusion and Exclusion Criteria

The patients included in this study met specific criteria, including high-quality OCT imaging with a central macular subfield thickness (CMST) of ≥300 μm, a best-corrected visual acuity (BCVA) between 24 and 72 letters on the ETDRS scale, an age ≥ 18 years, and a diagnosis of newly developed macular edema secondary to CRVO. The exclusion criteria consisted of corneal or lens opacities affecting OCT imaging, ocular diseases other than CRVO, the prior use of intraocular anti-VEGF or steroids, a history of vitreoretinal surgery in the study eye, or active intraocular inflammation such as uveitis. Since DEX exerts its effects through anti-inflammatory pathways, and inflammation-related systemic diseases can influence the macular edema outcomes, patients with systemic comorbidities other than hypertension (HT) or diabetes mellitus (DM) were excluded from this study. Specifically, patients with cardiovascular disease (other than HT), autoimmune diseases, chronic kidney disease, systemic vasculitis, or other chronic inflammatory disorders were not included. This restriction was applied to minimize the confounding effects of systemic inflammatory conditions on the treatment outcomes, and ensure a more homogeneous study population.

### 2.3. Assessment and Treatment Schedule

The baseline assessments included the collection of demographic data, such as age, gender, and systemic comorbidities, alongside comprehensive ophthalmological examinations at baseline and at the 3rd, 6th, and 12th months. These examinations included a BCVA measurement using ETDRS charts, an intraocular pressure (IOP) measurement with Goldmann applanation tonometry, and spectral-domain OCT (Cirrus HD-OCT 5000, Carl Zeiss Meditec AG, Inc., Dublin, CA, USA) to evaluate CMST and vitreoretinal interface changes. Colored fundus and fundus fluorescein angiography images were used to determine the fundus angiographic features and determine the need for a laser (TRC-50DX, Topcon Corporation, Tokyo, Japan). Spectral-domain OCT scans were used at each follow-up visit to monitor CMST changes and assess the treatment efficacy, providing critical guidance for therapeutic adjustments. High-resolution OCT imaging provided essential insights into the macular architecture, ensuring a precise evaluation of the treatment response and guiding adjustments. Those with an OCT signal strength above 7/10 were included in the study. The foveal center on the OCT fundus image was identified using an automated fovea localization algorithm. The examination protocol consisted of a 6 × 6 mm macular cube, centered on the fovea, composed of 128 horizontal b-scans of 512 a-scans each. The retinal thickness values were automatically calculated, using the Cirrus OCT software (version 8.1.0.117), for each of the nine areas corresponding to the ETDRS. The macular thickness analysis module in the Cirrus OCT system was used to determine the central macular thickness by referencing the internal limiting membrane (ILM) and the posterior boundary of the retinal pigment epithelium (RPE). To ensure accuracy, the segmentation lines were reviewed thoroughly, and necessary manual adjustments were made in cases where algorithmic errors were detected.

All the patients received an initial loading phase of three consecutive monthly anti-VEGF injections. Following the loading phase, the risks and benefits of corticosteroid therapy were explained to the patients, and their treatment was adjusted according to their group allocation. In Group 1 (early switch), the patients transitioned to DEX implant therapy after the loading phase, while in Group 2 (late switch), anti-VEGF therapy was continued with PRN dosing until six months, followed by a transition to DEX implant therapy.

### 2.4. Outcome Measures

The main outcome assessed was the variation in BCVA (ETDRS letters) at the 3rd, 6th, and 12th months. The secondary outcomes included OCT-derived metrics, such as CMST changes, which were used to quantitatively assess therapeutic response and anatomical improvements over time. The secondary outcomes also included the proportion of eyes that achieved a ≥15-letter gain in BCVA, the number of anti-VEGF and DEX injections administered, and the incidence of adverse events such as IOP elevation (≥5 mmHg) or cataract formation.

### 2.5. Statistical Analysis

A statistical analysis was performed using SPSS (version 25.0; IBM, Chicago, IL, USA). Data normality was assessed using the Shapiro–Wilk test. Descriptive statistics were presented as the mean ± standard deviation (SD) or the median (interquartile range, IQR) for continuous variables, and as percentages for categorical variables. Between-group comparisons of continuous variables were analyzed using an independent *t*-test for normally distributed data, or the Mann–Whitney U test for non-normally distributed data. Within-group changes over time were analyzed using repeated-measures ANOVA for normally distributed data, or Friedman tests for non-normally distributed data. Categorical variables were compared using the chi-square test or Fisher’s exact test where appropriate. CMST changes were analyzed using repeated-measures ANOVA for normally distributed data, or Friedman tests for non-normally distributed data. Correlations between BCVA improvements and OCT-derived CMST changes were assessed using Pearson or Spearman correlation coefficients, depending on the data distribution. A *p*-value < 0.05 was considered statistically significant for all the analyses.

## 3. Results

### 3.1. Baseline Characteristics

The baseline characteristics of the study groups were comparable, with no significant differences observed in the key parameters (*p* > 0.05). The mean age of the patients in Group 1 (early switch) was 67.8 ± 5.2 years, compared to 69.2 ± 6.2 years in Group 2 (late switch) (*p* = 0.214). The gender distribution was balanced between the groups, with 51.6% females in Group 1 and 43.3% in Group 2 (*p* = 0.517). Both groups had a similar lens status, with the majority of patients being phakic (*p* = 0.561). The baseline BCVA and CMST were also comparable between the groups, with a mean BCVA of 40.3 ± 21 ETDRS letters in Group 1 and 41.6 ± 21.5 letters in Group 2 (*p* = 0.822), and a mean CMST of 493.2 ± 76.2 μm in Group 1 versus 497.2 ± 122.9 μm in Group 2 (*p* = 0.470). These findings suggest that the groups were well matched at the start of the study. Details are provided in Table 1.

Regarding systemic comorbidities, HT and DM were the most common conditions among the study population. In Group 1, 41.9% had HT, 16.1% had DM, and 32.3% had both DM and HT. In Group 2, 46.7% had HT, 6.7% had DM, and 36.7% had both DM and HT. Patients with systemic inflammatory diseases, autoimmune disorders, chronic kidney disease, or other significant cardiovascular conditions (excluding HT) were excluded from the study to maintain a more homogeneous patient population and minimize the confounding effects of systemic diseases on the macular edema outcomes.

The smoking status of the patients was also evaluated. In Group 1, 32.3% (*n* = 10) of the patients were active smokers, 22.6% (*n* = 7) were former smokers, and 45.1% (*n* = 14) were non-smokers. Similarly, in Group 2, 30% (*n* = 9) were active smokers, 26.7% (*n* = 8) were former smokers, and 43.3% (*n* = 13) were non-smokers. There was no statistically significant difference in smoking status between the two groups (*p* = 0.774).

### 3.2. Visual Acuity (BCVA)

**Figure 1:**
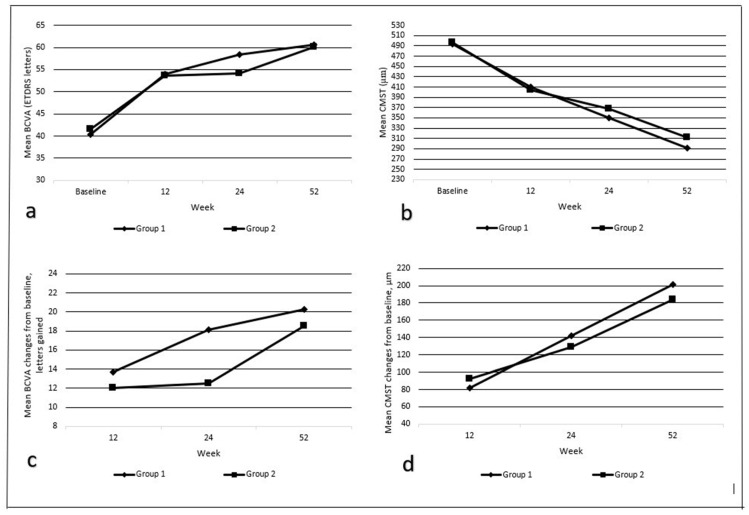
Changes in mean BCVA ETDRS letters and CMST values compared to baseline at 12th week, 24th week, and 52nd week. (**a**) Mean BCVA ETDRS letter change chart; (**b**) mean CMST chart; (**c**) mean BCVA ETDRS letter gain chart; and (**d**) mean anatomical gain changes from baseline. (BCVA, best-corrected visual acuity; CMST, central macular subfield thickness; ETDRS, Early Treatment Diabetic Retinopathy Study; DEX, dexamethasone.

Both groups demonstrated significant improvements in BCVA over the 12-month study period (Figure 1). At the 52nd week, Group 1 showed a mean gain of 20.3 ± 16.7 ETDRS letters, while Group 2 exhibited a mean gain of 18.5 ± 16.6 ETDRS letters, with no statistically significant differences between the groups (*p* = 0.885). A gain of ≥15 ETDRS letters was achieved in 51.6% (*n* = 16) of the Group 1 and 56.7% (*n* = 17) of the Group 2 patients.

The within-group comparisons from Table 2 show significant improvements in BCVA at all the follow-up visits (12th, 24th, and 52nd weeks) compared to the baseline in both Group 1 (early switch) and Group 2 (late switch) (*p* < 0.05 for all comparisons). However, the between-group comparisons from Table 3 reveal no significant differences in BCVA improvements at any of the follow-up visits between the two groups (*p* > 0.05). The BCVA improvements were supported by the OCT findings, which demonstrated significant reductions in CMST, correlating with improved visual outcomes.

### 3.3. Central Macular Subfield Thickness (CMST)

The OCT measurements provided high-resolution insights into CMST changes, which served as a key indicator of therapeutic efficacy. Both groups exhibited significant reductions in CMST from the baseline at all the follow-up visits (Figure 1). At the 52nd week, Group 1 had a mean reduction of 201.5 ± 82.6 μm, while Group 2 showed a reduction of 184.4 ± 121.4 μm, with no statistically significant differences between the groups (*p* = 0.121). These findings are summarized in Table 3.

In both groups, significant reductions in CMST were observed at the 12th, 24th, and 52nd weeks compared to the baseline, as shown in Table 2 (*p* < 0.05 for all time points). The between-group comparisons in Table 3 indicate no significant differences in CMST reductions between Group 1 and Group 2 at any of the follow-up visits (*p* > 0.05).

### 3.4. Intraocular Pressure (IOP)

The within-group comparisons shown in Table 2 reveal that, in Group 1 (early switch), a statistically significant increase in IOP was observed at the 24th week compared to the baseline (*p* < 0.05). In Group 2 (late switch), the IOP increased significantly at the 52nd week compared to the baseline (*p* < 0.05). These findings suggest that IOP changes were more prominent approximately three months after the administration of the DEX implant in both groups.

The between-group comparisons, as shown in Table 3, demonstrate a significant difference in IOP changes at the 52nd week, with Group 2 showing higher IOP values compared to Group 1 (*p* < 0.05). Additionally, at the 12th week, Group 2 exhibited a greater IOP change compared to Group 1, but this difference was not sustained at subsequent time points.

These results indicate that DEX implants are associated with a notable increase in IOP, which is typically observed around three months post-injection. Early switching (Group 1) appeared to mitigate late IOP elevations, while late switching (Group 2) was associated with more pronounced IOP changes at later follow-ups.

### 3.5. Treatment Burden

The patients in Group 1 required fewer anti-VEGF injections (mean: 3.7 ± 0.68) compared to Group 2 (mean: 5.8 ± 0.93, *p* < 0.001). However, Group 1 received more DEX injections (mean: 2.26 ± 0.45 vs. 1.5 ± 0.51, *p* < 0.001). The reduced treatment burden in the early-switch group aligns with the enhanced ability of OCT to monitor macular edema resolution with fewer injections. These data are presented in Table 3.

### 3.6. Key Observations

- Both early and late switching from anti-VEGF to DEX implants effectively improved BCVA and reduced CMST.
- Group 1 (early switch) had a slightly greater reduction in CMST and fewer anti-VEGF injections, suggesting a lower treatment burden.
- Group 2 (late switch) exhibited a higher risk of IOP elevation at the 52nd week.
- The key findings underscore the pivotal role of OCT in guiding early-switching decisions, enabling clinicians to optimize treatment strategies and reduce the burden without compromising efficacy.

## 4. Discussion

This study compared the outcomes of early versus late switching from anti-VEGF therapy to DEX implants in treatment-naïve patients with macular edema secondary to CRVO. The findings align with the existing literature, while contributing new insights into the timing of therapy adjustments.

Both the early- and late-switch groups showed significant improvements in BCVA, which is consistent with prior studies highlighting the efficacy of anti-VEGF and DEX implants for the treatment of CRVO-related macular edema. For instance, the GENEVA study reported significant visual gains within two months of DEX implant administration, with sustained improvements at six months [10]. Similarly, the SCORE2 trial demonstrated comparable BCVA improvements with anti-VEGF therapy, showing mean gains of 16 letters at one year [21].

In this study, the BCVA improvements were slightly greater in the early-switch group at 52 weeks, likely due to the earlier initiation of corticosteroid therapy, which may have effectively controlled inflammation and vascular leakage (Table 2 and Table 3). This finding aligns with research suggesting that corticosteroids can provide additional advantages in reducing edema and stabilizing the retina when introduced early [16]. Conversely, prolonged anti-VEGF therapy before transitioning to corticosteroids may delay these benefits, emphasizing the importance of timely intervention in optimizing the visual outcomes of CRVO treatment. According to the results of a systematic meta-analysis study by Hu et al., who investigated the results of anti-VEGF and DEX implant treatments by examining the results of five randomized controlled trials, no significant differences were found between these two treatments, and both treatments had similar therapeutic effects in the treatment of ME associated with RVO [22]. Although both treatment methods have similar effects in the management of the disease separately, there is a gap in the literature regarding when to switch to the DEX treatment in patients who still have ME after the initial anti-VEGF loading. The current study found that early or late switching did not make a significant difference in functional gain, but the early-switch group demonstrated a slightly better gain (Table 2 and Table 3).

Both groups demonstrated significant reductions in CMST, supporting the established role of anti-VEGF and DEX therapies in mitigating macular edema. A meta-analysis comparing these modalities concluded that both therapies are effective at reducing retinal thickness, with DEX implants offering a longer duration of effect [23]. Early switching in the current study resulted in slightly greater CMST reductions at 52 weeks compared to late switching, although this difference was not statistically significant (Table 2 and Table 3). This trend aligns with studies suggesting that combining anti-VEGF and DEX therapies sequentially may enhance anatomical outcomes by targeting different aspects of edema pathophysiology [24].

This study further supports the critical role of OCT in guiding the timing of therapeutic transitions. OCT imaging not only enables precise measurement of CMST, but also provides insights into subtle changes in retinal anatomy, which can inform individualized treatment plans. Previous research has highlighted the benefits of early intervention with DEX implants for macular edema that persists despite anti-VEGF therapy. For instance, a real-world study by Al Ajmi et al. demonstrated that timely switching to DEX implants significantly improved anatomical and functional outcomes in patients with refractory macular edema, particularly when OCT findings guided the decision-making processes [25]. Similarly, Martins et al. emphasized the alignment between real-world outcomes and clinical trial data, reinforcing the utility of DEX implants as an effective alternative in cases of a suboptimal response to anti-VEGF therapy [26]. These findings underscore the importance of OCT as a decision-making tool, enabling clinicians to optimize the timing of therapeutic interventions and reduce the overall treatment burden. In the current study, high-resolution OCT was instrumental in monitoring treatment responses and guiding follow-up decisions, demonstrating its pivotal role in achieving precise and effective management outcomes (Table 2 and Table 3).

The timing of DEX implant administration significantly influenced IOP changes (Table 3). In Group 1 (early switching), IOP increases were observed at 24 weeks, approximately three months after the initial DEX injection. This is consistent with the known pharmacokinetics of DEX implants [10]. In Group 2 (late switching), the IOP increases were delayed, occurring at the 52nd week, following the transition to corticosteroids. Between-group comparisons revealed that late switching was associated with higher IOP values at 52 weeks, highlighting the importance of monitoring IOP, particularly in patients with delayed steroid exposure.

These findings align with prior reports indicating that IOP elevations occur in approximately 15–20% of eyes treated with DEX implants, peaking at around three months post-injection [15]. Although generally manageable with topical therapy, such side effects underscore the need for individualized risk assessment when selecting treatment regimens.

The early-switch group required fewer anti-VEGF injections compared to the late-switch group (Table 3), which is consistent with previous studies indicating that transitioning to DEX therapy can reduce the frequency of injections without compromising efficacy [14]. However, this benefit came at the cost of an increased exposure to corticosteroids, potentially elevating the risk of side effects such as IOP elevation and cataract formation. Balancing the trade-off between treatment burden and side effect risks remains a critical consideration in CRVO management.

This study holds significant value as a real-world, retrospective analysis, reflecting the challenges and outcomes encountered in daily clinical practice. Unlike tightly controlled randomized clinical trials, the design of this study allowed for the inclusion of a diverse patient population and real-life variables, providing a practical perspective on treatment strategies for CRVO-related macular edema. The use of high-resolution OCT was integral to the study, enabling the precise monitoring of treatment responses and facilitating individualized decision-making. The findings highlight the potential benefits of early switching to dexamethasone implants, particularly in reducing the treatment burden while maintaining comparable efficacy to late switching. Moreover, the real-world nature of this study underscores its relevance for guiding clinical decision-making, especially in resource-limited settings where frequent anti-VEGF injections may not be feasible. These attributes enhance the practical applicability of the results, making them valuable for routine ophthalmological practice.

This study has several strengths. Firstly, it included a well-defined cohort of treatment-naïve CRVO patients, ensuring homogeneity in the baseline characteristics. Statistical analysis confirmed no significant differences between the groups in baseline demographic and clinical characteristics, supporting group homogeneity (Table 1). The real-world nature of this study adds significant value by reflecting the challenges and variables encountered in routine clinical practice. The standardized follow-up protocols and the inclusion of both visual (e.g., BCVA) and anatomical (e.g., CMST) outcomes, assessed using advanced OCT imaging, provided a comprehensive evaluation of the treatment effects. Furthermore, the practical benefits of early-switching strategies in reducing the treatment burden were demonstrated under real-world conditions. OCT played a pivotal role in this process, as it facilitated the accurate tracking of macular thickness changes and guided timely therapeutic adjustments, enhancing the overall treatment precision and effectiveness.

However, this study also has some limitations. The retrospective design limited causal inference and introduced the potential for selection bias. Additionally, the relatively small sample size may reduce the generalizability of the findings. The lack of patient-reported outcomes, such as vision-related quality of life, represents another limitation, as it prevented a full assessment of the patient-centered impact of treatment. Future prospective studies should explore the integration of OCT-derived biomarkers for the more precise timing of therapeutic transitions. Despite these constraints, this study provides valuable insights into the timing and outcomes of therapy transitions for CRVO-related macular edema in a real-world setting.

## 5. Conclusions

Both early and late switching from anti-VEGF to DEX implants showed comparable efficacy in improving the visual (BCVA) and anatomical (CMST) outcomes in CRVO patients. Early switching, guided by high-resolution OCT findings, offered slightly greater gains and a reduced treatment burden, making it particularly beneficial in real-world settings, especially for patients with limited adherence to frequent injections. These findings emphasize the value of OCT in optimizing individualized treatment strategies, and highlight the need for further research to refine transition timing and enhance outcomes in macular edema management.

## Figures and Tables

**Table 1:** Baseline demographic and ocular characteristics of study participants.

	Group 1 (*n*: 31)	Group 2 (*n*: 30)	*p*-Value
Baseline Parameters			
Average age ± SD, years (median, IQR 25th–75th)	67.8 ± 5.2 (68, 66–71)	69.2 ± 6.2 (69, 66–74)	0.214 ^a^
Gender, *n* (%)			0.517 ^b^
Female	16 (51.6)	13 (43.3)	
Male	15 (48.4)	17 (56.7)	
Lens status, *n* (%)			0.561 ^b^
Phakic	24 (77.4)	25 (83.3)	
Pseudophakic	7 (22.6)	5 (16.7)	
Central macular subfield thickness ± SD, µm (median, IQR 25th–75th)	493.2 ± 76.2	497.2 ± 122.9 (480, 430–520)	0.470 ^a^
BCVA score ± SD (ETDRS letters) (median, IQR 25th–75th)	40.3 ± 21 (39, 24–54)	41.6 ± 21.5 (39, 24–54)	0.822 ^a^
Intraocular pressure ± SD, mmHg (median, IQR 25th–75th)	15.5 ± 4.4 (15, 12–19)	15.1 ± 2.9 (14, 13–18)	0.890 ^a^
Systemic comorbidities, *n* (%)			0.716 ^b^
Diabetes mellitus	5 (16.1)	2 (6.7)	
Hypertension	13 (41.9)	14 (46.7)	
Diabetes mellitus and hypertension	10 (32.3)	11 (36.7)	
Smoking status, *n* (%)			0.773 ^b^
Active smokers	10 (32.3)	9 (30)	
Former smokers	7 (22.6)	8 (26.7)	
Non-smokers	14 (45.1)	13 (43.3)	

SD: standard deviation; BCVA: best-corrected visual acuity; ETDRS: Early Treatment Diabetic Retinopathy Study; DEX: dexamethasone. Values in table are presented as mean ± SD, while for nonparametric distributions, median values with interquartile ranges (25th–75th percentiles) are additionally provided. Statistical significance was set at *p* < 0.05 within 95% confidence interval for comparisons between Group 1 and Group 2. Statistical analyses were conducted using Mann–Whitney U test (^a^) and chi-square test (^b^).

**Table 2:** Intra-group changes in BCVA and CMST throughout study period.

	Group 1 (*n*: 31)				Group 2 (*n*: 30)			
BCVA (ETDRS Letters)	Mean ± SD (Median, IQR 25–75)	95% CI of Mean Lower/Upper	*p*-Value	95% CI of Difference, Lower/Upper	Mean ± SD (Median, IQR 25–75)	95% CI of Mean, Lower/Upper	*p*-Value	95% CI of Difference, Lower/Upper
Baseline	40.3 ± 21 (39, 24–54)	32.6/48			41.6 ± 21.5 (39, 24–54)	29.5/45.6		
12th week	54 ± 19.9 (52, 37–74)	46.7/61.4	<0.001 ^a^	−19.5/−7.9	53.6 ± 21.5 (63, 34–72)	44.1/60	<0.006 ^a^	−18.8/−5.2
24th week	58.4 ± 17.6 (63, 54–76)	51.9/64.8	<0.001 ^a^	−24.1/−12	54.1 ± 27 (70, 23–75)	40.6/60.4	<0.002 ^a^	−19.6/−5.3
52nd week	60.6 ± 18.2 (62, 55–72)	53.9/67.3	<0.001 ^a^	−26.4/−14.2	60.1 ± 16.2 (64, 42–71)	53.5/63.5	<0.001 ^a^	−24.7/−12.3
CMST, μm								
Baseline	493.3 ± 76.2	465.2/521.1			497.2 ± 122.9 (480, 422–530)	353.4/472.6		
12th week	410.6 ± 51.6	391.7/429.5	<0.001 ^b^	60.7/104.4	404.8 ± 60.2	285.4/364.1	<0.001 ^a^	56.7/128.1
24th week	350.6 ± 43.9	334.5/366.7	<0.001 ^b^	113.3/171.8	367.5 ± 51.9 (360, 338–387)	269.8/355.9	<0.001 ^a^	85.2/174.1
52nd week	291.7 ± 36.2	278.4/305	<0.001 ^b^	171.2/231.7	312.8 ± 40.9 (297, 285–343)	249.2/302.9	<0.001 ^a^	139.1/229.7
IOP, mm HG								
Baseline	15.5 ± 4.4 (15, 13–20)	13.9/17.2			15.1 ± 3 (15, 14–19)	14/16.2		
12th week	16.4 ± 5 (16, 14–20)	14.6/18.3	0.169 ^a^	−2.5/0.7	15.9 ± 2.4 (16, 15–19)	14.9/16.7	0.146 ^a^	−1.8/0.4
24th week	18 ± 4.2	16.5/19.5	<0.001 ^a^	−3.8/−1.1	15.9 ± 2.1 (16, 15–19)	15.3/16.8	0.125 ^a^	−2.2/0.4
52nd week	16.6 ± 3.5	15.3/17.9	0.121 ^a^	−2.5/0.4	19.2 ± 1.3	18.7/19.6	<0.001 ^a^	−4.9/−3.2

SD: standard deviation; BCVA: best-corrected visual acuity; CMST: central macular subfield thickness; ETDRS: Early Treatment Diabetic Retinopathy Study; IOP: intraocular pressure; CI: confidence interval. Values in table are presented as mean ± SD, while for nonparametric distributions, median values with interquartile ranges (25th–75th percentiles) are additionally provided. Statistical significance was set at *p* < 0.05 within 95% confidence interval for comparisons between baseline and follow-up visits. Statistical tests applied included Wilcoxon signed-rank test (^a^) for paired nonparametric data, and paired *t*-test (^b^) for normally distributed paired comparisons.

**Table 3:** Comparative analysis of BCVA (ETDRS letters) and CMST changes between groups across different follow-up visits.

	Group 1 (*n*: 31)		Group 2 (*n*: 30)		
BCVA Changes (ETDRS Letters)	Mean ± SD (Median, IQR 25–75)	95% CI of Mean, Lower/Upper	Mean ± SD (Median, IQR 25–75)	95% CI of Mean, Lower/Upper	*p*-Value
12th week	13.7 ± 15.8 (8, −2.5–26)	8/19.5	12 ± 18.2	5.2/18.8	0.447^a^
24th week	18.1 ± 16.4 (16, 9–21)	12/24.1	12.5 ± 19.2 (14, −5–21)	5.3/19.6	0.157 ^a^
52nd week	20.3 ± 16.7 (17, 7–35)	14.2/26.4	18.5 ± 16.6	12.3/24.7	0.885 ^a^
CMST changes (µm)					
12th week	82.6 ± 59.5 (77, 30–140)	60.7/104.4	92.4 ± 95.6 (60, 34–121)	43,8/132.6	0.801 ^a^
24th week	142.6 ± 79.8	113.3/171.8	129.7 ± 119 (117, 40–175)	58.3/141.9	0.267 ^a^
52nd week	201.5 ± 82.6	171.2/231.7	184.4 ± 121.4 (158, 109–233)	76.9/196.9	0.121 ^a^
IOP changes (mmHG)					
12th week	0.9 ± 4.3	−0.7/2.5	0.7 ± 3	−0.4/1.8	0.855 ^b^
24th week	2.5 ± 3.7	1.1/3.8	0.9 ± 3.5 (2.1, −2.1–4.1)	−0.4/2.2	0.101 ^a^
52nd week	1.1 ± 4	−0.4/2.5	4 ± 2.3 (4.2, 2.3–6.3)	3.1/4.9	0.002 ^a^
Number of injections of DEX ± SD	2.26 ± 0.45 (2, 2–3)	2.1/2.4	1.5 ± 0.51 (1.5, 1–2)	1.3/1.7	<0.001 ^a^
Number of injections of anti-VEGF ± SD	3.7 ± 0.68 (4, 3–4)	3.5/4	5.8 ± 0.93 (6, 5–6)	5.5/6.2	<0.001 ^a^

SD: standard deviation; BCVA: best-corrected visual acuity; CMST: central macular subfield thickness; ETDRS: Early Treatment Diabetic Retinopathy Study; DEX: dexamethasone; anti-VEGF: anti-vascular endothelial growth factor; CI: confidence interval. Values in table are presented as mean ± SD, while for nonparametric distributions, median values with interquartile ranges (25th–75th percentiles) are additionally provided. Statistical significance was set at *p* < 0.05 within 95% confidence interval for comparisons between Group 1 and Group 2. Statistical analyses included Mann–Whitney U test (^a^) for nonparametric variables and independent samples *t*-test (^b^) for normally distributed independent comparisons.

## Data Availability

The data presented in this study are available from the corresponding author upon request. The data are not publicly available, due to ethical restrictions.
